# Risks with Gynaecological problems on the health of University Students

**DOI:** 10.12669/pjms.35.3.834

**Published:** 2019

**Authors:** Rukiye Hobek Akarsu, Selda Yuzer Alsac

**Affiliations:** 1 *Dr. Rukiye Hobek Akarsu, Department of Midwifery, Faculty of Health Sciences, Yozgat Bozok University, Yozgat, Turkey*; 2 *Dr. Selda Yuzer Alsac, Department of Nursing, Faculty of Health Sciences, Yozgat Bozok University, Yozgat, Turkey*

**Keywords:** Young girls, Risk with gynecological problems, University female students

## Abstract

**Objective:**

To determine the risk with gynecological problems on the health of female university students.

**Methods:**

The study was conducted as a descriptive in university in Central Anatolia, Turkey. The research population was composed of 1305 female university students studying at a university in Central Anatolia. The study was conducted between January and March 2017. The data were collected through a questionnaire consisting of 23 questions prepared by the researchers to determine the socio-demographic characteristics of the participants and the risk with gynecological problems they encountered. Numerical and percentage statistics were used to analyze the data.

**Results:**

About 65.4 percent of the female students in this study previously had gynecological examination, and 38.8 percent of them were diagnosed with gynaecological problems. It was found that 87.6 percent of the female students had risk with gynecological disorders. the most common ones being dysmenorrhoea (63.2%), premenstrual syndrome (56.7%), urinary tract infection (22.4%), and polycystic ovarian syndrome (13%), respectively.

**Conclusions:**

It was found that nearly all the young girls had risk with gynecological problems and nearly half of them were diagnosed with different gynaecological disorders.

## INTRODUCTION

The World Health Organization defines the 10-19 age group as the “Adolescent” group and the 15-24 age group as the “Young” group. Due to the intersection of adolescent and youth ages, the 10-24 age group is considered as “Young People”.^1^In Turkey, the share of the 10-24 age group within the total population is 21.1%. This rate is fairly high, meaning that one in every five people is in the youth age group.^2^ During the youth period, which is generally considered as the transition period from childhood to adulthood.^3^Young peoples experience not only changes in brain, neuro-endocrine system and hormone concentrations but also physical and emotional changes and risks with gynecological problems that affect reproductive health and social life.^4^Adolescents encounter some risks with gynecological problems such as menstrual problems, vaginal discharge and infections, pelvic mass and ovarian cysts, trauma and sexual abuse, genital system anomalies, abdominal and pelvic pain, adolescent pregnancies, breast problems, and early and late puberty.^5^ In a study 33.4% of the adolescents consulted a doctor with complaints of menstrual disorder and that 2.8 percent of all the patients were hospitalized for treatment.^6^

Furthermore, as revealed by the study, the young population is generally perceived as a healthy group that does not require health care services, and thus they cannot adequately benefit from these services.^7^ For this reason, the diagnosis of the gynecological problems whose onset is rooted in this period may be delayed, leading to higher mortality or morbidity at adult ages. However, it must be noted that the majority of the health problems experienced in this period may be prevented.^8^

It is important for young womens to receive appropriate health care so that they can cope with the gynecological problems they may experience during this period. Also, it is important to know the risks with gynecological problems that can be encountered frequently so that effective interventions can be made to prevent these problems.^5^

Early detection and treatment of problems and taking the necessary precautions against the problems improve both the health level and life quality of young women. Healthy young womens can contribute to community health by increasing women’s health.

There are few studies on risk with gynecological problems in young women in the world, or a limited number of studies that determine the prevalence of extensive risk with gynecologic problem. This study aimed to determinate the risk with gynecological disorders in female university students.

## METHODS

We conducted a descriptive study in university in Central Anatolia, Turkey. The research population was composed of female university students studying at a university in Central Anatolia. It was planned to include all female students in the university which were 1612 in number..

### Inclusion Criteria

- Being 18 or older
- Accepting to participate in the study
- Continuing undergraduate education

### Exclusion criteria

- Under 18 years old
- Having communication problems
- Having physical disabilities

The study was completed with 1305 female students with a response rate of 80.9% of the universe was reached.

The data was collected with a questionnaire, consisting of 23 questions aimed to determine the age of the participants, the place they live in and the gynecological problems they have. The most common gynecological problems were listed and the definition and symptoms of each problem was given in parenthesis. For example, Premenstrual Syndrome (a condition characterized by some emotional changes that start a few days before menstruation and which could continue throughout the menstruation such as edema, headache, nervousness and tension), or urinary tract infection (a condition characterized by foul discharge, inguinal pain, and urinary burning). Gynecological problems diagnosed by a doctor were investigated separately, e.g., physician-diagnosed polycystic ovarian syndrome. The questionnaire was distributed among the students selected from the faculties or schools based on the simple random numbers table during the lesson breaks. The data was entered into a computer and numerical and percentage statistics were used to analyze the data.

The participants were informed about the aim of the study and their oral and written consent was obtained by asking them to sign the participant consent Form. The written permission was received from Bozok University and the ethical permission was obtained from Bozok University Clinical Sciences Ethical Committee. (Ethical committee registration number: 38812373-050.01/1).

Statistical Package for Social Science (SPSS) 21 was used to analyze the data. The Kolmogorov-Smirnov test and Shapiro-Wilk test were used to determine the normal distribution of the data. Descriptive statistics (percentage, average, and standard deviation) (p< .05)

## RESULTS

Socio-demographic data of the female students in the study is given in Table-I. The average age of the students was 19.7±0.45, and the average of Body Mass Index (BMI) was found to be 21.4±3.1. 99.2 percent of the students were single; 66.7% of them perceived their economic situation as good; and 64.6% of them stayed in the state dormitory.

**Table-I:** Socio-demographic characteristics of the young womens.

Socio-demographic characteristics	n	%
Age	19.7±0.45	
BMI	21.4±3.1	
***Marital Status***		
Married	11	0.8
Single	1294	99.2
***Economic Status***		
Good	359	27.5
Average	857	66.7
Bad	89	6.9
***Place of Residence***		
Village	270	207
Town	411	31.5
City	624	47.8
***Accommodation***		
State Dormitory	843	64.6
Private Dormitory	329	25.2
House	133	10.2

Total	1305	100.0

Health behaviors of the female students in our study is shown in Table-II. It was found that 90.4% of the students did not smoke, and 98.5% of the students stated that they do not use alcohol. The study further found that 75.8% of the participants do not do regular exercise.

**Table-II:** Health behaviors of the young womens in the study.

Health behaviors	n	%
***Smoking***		
Smoker	125	9.6
Non-smoker	1180	90.4
Number of Cigarettes^*^ (day)	9.03±8.7	
***Alcohol consumption***		
Yes	19	1.5
No	1286	98.5
***Doing Exercise***		
Yes	316	24.2
No	989	75.8
Frequency of Doing Exercise**		
Everyday	78	24.7
Occasionally	133	42.0
Once a week	48	15.2
Twice a week and more	57	18.0
***Mostly preferred food types***		
Legume	575	44.1
Vegetable- Fruit	469	35.9
Meat and Meat Products	261	20.0

Total	1305	100

* percentages (n=125),

** percentages (n=316)

The gynecological characteristics of the female students in this study are given in (Table-III. The average menstrual age of the participants was found to be 13.5±1.31. About 71.6 percent of the participants stated that their menstrual cycle was regular, 65.4% of the female students in this study further stated that they had a gynecological examination before and 38.8% reported that they had a diagnosed gynecological problem. Furthermore, 83.4% of the participants believed that it is necessary to have regular gynecological examination and 46.9% of them believed that examination should be once in every six months. Moreover, our study revealed that 69.2% of the female students do not know breast self-examination (BSE), that 82.8% of those who know BSE do not do it, that 9.7% of the students had gynecological cancer history in their family, and that 69.8% of those who had gynecological cancer also had breast cancer.

**Table-III:** Gynecological characteristics of the young womens.

Gynecological Characteristics	*n*	%
Menstrual age	13.5±1.31	
***Having regular menstrual cycles***		
Regular	934	71.6
Irregular	371	28.4
***Time between the menstrual cycles***		
Less than 28 days	383	29.3
Between 28-34 days	796	61.0
35 days and more	126	9.7
Length of menstrual periods	5.7±1.4	
***Having a gynecological examination before***		
Yes	451	34.6
No	854	65.4
Having a gynecological Problem diagnosis*		
Diagnosed	175	38.8
Not diagnosed	276	61.2
***Opinions on having regular gynecological examination***		
Necessary	1088	83.4
Not necessary	217	16.6
Opinions on the frequency of having regular gynecological examination**		
Necessary once in every 6 months	510	46.9
Necessary once a year	449	41.3
Necessary when there is a health problem	129	11.9
***Knowing about BSE***		
Yes	402	30.8
No	903	69.2
Doing BSE***		
Yes	69	17.2
No	333	82.8
***Having Gynecological cancer in the family***		
Yes	126	9.7
No	1179	90.3
Type of the gynecological cancer in the family****		
Breast Cancer	88	69.8
Uterus Cancer	33	26.2
Ovarian Cancer	3	2.4
Cervix Cancer	2	1.6

Total	1305	100.0

* Percentages were taken over n=451,

** Percentages were taken over n=1088,

*** Percentages were taken over n=402,

**** Percentages were taken over n=126

The gynecological problems experienced by the female students in this study are shown in Table-IV. It was revealed that 87.6% of the participants had a gynecological problem. The most widespread gynecological problems were dysmenorrhoea (63.2%) premenstrual syndrome (PMS) (56.7%), urinary tract infection (22.4%) respectively.

**Table-IV:** Gynecological problems of the young womens.

Gynecological Problems	n	%
***Having a risk with gynecological problem***		
Yes	1143	87.6
No	162	12.7
Risk with Gynecological Problems*		
Dysmenorrhea	825	63.2
PMS	740	56.7
Urinary tract infection	299	22.4
Polycystic ovarian syndrome	170	13.0
Physician-diagnosed polycystic ovarian	46	3.5
Amenorrhea	145	11.1
Fungal infection	115	8.8
Bacterial infection	37	2.8
Parasitic infection	33	2.5
Genital Wart	30	2.3
Physician-diagnosed myoma	13	1.0
Physician-diagnosed pelvic inflammatory disease	11	0.8
Physician-diagnosed Breast Mass	10	0.8
Chronic Pelvic Pain	9	0.7

Total	1305	100.0

* More than one response was given to the question.

## DISCUSSION

The study showed that 86.7% of the young female students in this study were at risk of gynecological problem. Demir et al,^9^ has reported that regular exercise, reduction in caffeine and alcohol consumption, not smoking, regular and balanced diet, and consumption of fresh fruit and vegetable reduces the risk with gynecological problems. It was observed that alcohol and smoking rates are low. However increased exercise and balanced nutrition was also low. This situation may have increased the risk of gynecological problems.

This study revealed that 34.6% of female students had a gynecological examination due to a health problem and that 38.8% were diagnosed with a gynecological disorders About half of the students stated that it is necessary to have a gynecological examination every six months. Demiray et al.^10^ found that about one third of women, the majority of whom are aged between 31-40, have a gynecological examination. As gynecological problems are considered to be embarrassing in Turkey, the rate of seeing a gynecologist is low. This study also revealed that although half of the participants believe that a gynecological examination is necessary at least once in six months, only about one third of them had seen a gynecologist so far.

The study also showed that two thirds of the university students do not know how to do BSE and that only 17.2% of those who know BSE actually do it. Alpteker et al.^11^ has demonstrated that 19.7% of those who know how to do BSE do not actually do it. About half of the female students studying at university do not know BSE and the majority of those who know it do not do it.

It was also found that 10% of the participants had a family history of gynecological cancer and that 69.8% of these cancer were breast cancer. Although breast cancer was found to have a higher percentage compared to other cancer types, female students do not know BSE and those who know BSE do not do it, which is a striking finding. This may be attributed to the fact that woman breast is perceived as the symbol of youth and sexual attraction, and thus it is a sin, shame, and a taboo to touch it.

Furthermore, it was revealed that almost all the female students in the study had at least one gynecological problem. The gynecological problems experienced vary, the most common ones being dysmenorrhoea (63.2%) and PMS (56.7%). Previous studies have also found the prevalence of dysmenorrhoea to be between 52.07% and 86.9% and PMS to be between 8.75% and 85%.^12^-^15^

The study conducted in Turkey to determine the gynecological problems revealed that the majority of the adolescents who had the dysmenorrhoea problem also had PMS.^9^As revealed by this study, the prevalence of PMS and dysmenorrhoea is close to each other, implying that young womens may be experiencing these problems at the same time.

We found that 22.4% of the female students in our study had the symptoms of urinary tract infection. Despite the detailed literature review, we could not find any recent studies conducted to determine the prevalence of urinary tract infection problem in young womens. However, the studies carried out with young womens and adolescents found the prevalence of urinary tract infection to be between 2% and 30 percent.^16^-^18^ The study also revealed similar findings as 14.1% of the students had infections such as fungus infection (8.8%), bacterial infection (2.8%), and parasitic infection (2.5%). Moreira et al.^19^ found bacterial (5%) and fungus infection (4%) in adolescents. The study findings coincide with those in the literature.

The rate of physician-diagnosed polycystic ovarian syndrome (POS) was found to be 3.5%. In another study, the prevalence of diagnosed POS was found to be 2.6% in women aged 25-34.^20^ In the literature, the prevalence of POS in women at reproductive age was found as 4 to 10 percent.^21^,^22^ In this study, the rate of the young womens who were not given a diagnosis by a physician, but who had POS symptoms was found to be 13%. It is believed that this rate may increase when detailed examinations are conducted.

One of the important health problems in adolescent womens is the sexually transmitted diseases (STD). A study found that 24% of sexually active adolescent womens had STD, the most common one being human papilloma virus (HPV) infection.^23^,^24^ In this study, 2.3% of the womens stated that they had the symptoms of genital wart. The number of studies conducted in Turkey on STD is limited. As revealed by this study, the rate of HPV in Turkey is low compared to the other countries in the literature. This may be attributed to the fact that in societies where sex is associated with marriage, the rate of adolescents with active sex life is low.

## CONCLUSION

It was revealed that young womens in this study had high risk of gynecological disorders. Risk with gynecological problems can lead to worse health conditions. It is suggested that reproductive health and risk with gynecological problems for young women should be initiated in the pre- adolescence period and that the university health education programs should be organized to cover interventions to prevent risk with gynecological disorders. It is recommended that prevalence studies specific to risk with gynecological problems common in young womens be conducted with larger samples and different populations.

### Author`s Contribution

**RHA** conceived, designed and did statistical analysis & editing of manuscript & did review and final approval of manuscript.

**SYA** did data collection and manuscript writing.
